# Pulsed TIG Cladding of a Highly Carbon-, Chromium-, Molybdenum-, Niobium-, Tungsten- and Vanadium-Alloyed Flux-Cored Wire Electrode on Duplex Stainless Steel X2CrNiMoN 22-5-3

**DOI:** 10.3390/ma16134557

**Published:** 2023-06-24

**Authors:** Daniel Mutașcu, Olimpiu Karancsi, Ion Mitelea, Corneliu Marius Crăciunescu, Dragoș Buzdugan, Ion-Dragoș Uțu

**Affiliations:** 1 Department of Materials and Fabrication Engineering, Politehnica University Timisoara, Bulevardul Mihai Viteazul nr.1, 300222 Timisoara, Romania; daniel.mutascu@student.upt.ro (D.M.); ion.mitelea@upt.ro (I.M.); corneliu.craciunescu@upt.ro (C.M.C.); dragos.buzdugan@upt.ro (D.B.); 2 Department of Oral Implantology and Prosthetic Restorations on Implants, Victor Babeș University of Medicine and Pharmacy Timișoara, Eftimie Murgu Square, No. 2, 300041 Timișoara, Romania; karancsi.olimpiu@umft.ro

**Keywords:** pulsed current TIG welding, Corodur 65 alloy, duplex stainless steel

## Abstract

The hardfacing process aims to increase the life span of structural components in the petrochemical, mining, nuclear and automotive industries. During operation, these components are subject to demands of abrasion wear, cavitation erosion and corrosion. Duplex stainless steels are characterized by high mechanical characteristics and corrosion resistance, but poor behavior to abrasive wear and cavitation erosion. The improvement in wear resistance is possible by selecting and depositing a special alloy on the surface using a joining technique that ensures a metallurgical bonding between the layer and the substrate. The experimental investigations carried out in this work demonstrate the ability of the TIG pulsed welding process to produce layers with good functional properties for engineering surfaces. The “Corodur 65” alloy was deposited on a duplex-stainless-steel substrate, X2CrNiMoN22-5-3, using a series of process parameters that allowed for the control of the cooling rate and heat input. The properties of the deposited layers are influenced not only by the chemical composition, but also by the dilution degree value. Since the deposition of layers through the welding operation can be considered as a process with several inputs and outputs, the control of the input parameters in the process aims at finishing the granulation and the structure in the fusion zone as well as limiting the segregation phenomena. The aim of this work is to investigate the microstructural characteristics of the iron-based alloy layer, Corodur 65, deposited via pulsed current TIG welding on duplex X2CrNiMoN22-5-3 stainless-steel substrates.

## 1. Introduction

In most cases, the removal of a mechanical part from operation is mainly caused by the following three phenomena: wear, corrosion and fatigue [1,2,3]. They are localized in mechanical constructions, in the automobile and aeronautical industry, in the railway and weapon industry, etc. Estimates of the past 20 years made in the USA, Germany, France and Canada show that the direct expenses related to the replacement of parts degraded by these three phenomena represent approx. 5% of the national industries’ turnover [3].

Surface layers play an important role in the behavior of mechanical parts; in recent decades, we have been witnesses to a real revolution in the field of surface engineering techniques [4].

To limit the harmful effects of mechanical wear and cavitation erosion, a number of possible solutions were suggested without significant success [5,6]. Thus, some research focused on the deposition of protective coatings using thermal spraying, electric arc welding, PVD and CVD processes, thermochemical treatments, etc. [4,5,6].

Panadda Niranatlumpong et al. [7] studied the microstructure and wear resistance of NiCrBSi–WC and NiBSi–WC coatings deposited on the 304 SS substrate using electric arc spraying. It was highlighted that the microstructure of the NiCrBSi–WC coating consists predominantly of WC/W_2_C phases together with NiCr and NiCrW solid solutions. The microstructure of the NiBSi–WC coating consisted of WC/W_2_C phases together with Ni and NiW solid solutions. The absence of hard chromium carbide precipitates contributed to the lower hardness of the NiBSi-WC coating compared to the NiCrBSi–WC coating.

Thermal spraying processes are usually easy to conduct, carried out in a short time and, through the reduced thermal shock, they allow for the obtainment of layers with minor changes in the properties of the substrate. However, these layers show a series of drawbacks related to the presence of porosities, low adhesion to the substrate and inhomogeneous microstructure [8].

On the other hand, the deposition processes via electric arc welding ensure a good adhesion of the layer to the substrate, but the thermal shock induced in the material can cause unfavorable microstructural changes. Additionally, the dilution degree of the filler material with the base material can worsen the performance properties of the deposited layer [9,10,11,12].

The hardfacing techniques recommended to be performed using welding are plasma transferred arc welding (PTA), TIG welding, welding in shielding gas atmosphere (SMA) and laser beam welding [13,14]. Generally, Fe-, Ni- and Co-based alloys are selected for hardfacing.

Qian M. and colleagues [15] studied the effects of the powder feed rate (PFR) and translation speed (TS) of a laser beam being investigated by the following three nickel-based laser-clad hardfacing alloys: Colmonoy 6, Colmonoy 88 and AI-1236. The width-to-height ratio of the single clad pass and the dilution rate were found to decrease with the increasing PFR and reducing TS.

Rojacz and colleagues [16] analyzed the microstructural changes caused by the processing of complex-alloyed hardfacings and the resulting wear performance under impact–abrasive conditions. Beyond the chemical composition of welded hardfacings, processing parameters, such as heat input, interpass temperature, etc., have a crucial influence on the final tribological properties of the deposited coatings.

Singh S. and colleagues [17] analyzed the hardness and wear behavior of the surface hardfacing process using EN 353 alloy steel deposited via thermal spraying, TIG welding and electric arc welding. Due to the high cost of remanufacturing engineering components via welding, they recommend the hardfacing process.

The scientific paper [18] found that the toughness of tungsten carbides was a predominant factor in determining the high-stress abrasion resistance of Ni-WC hardfacing coatings. Cemented tungsten carbide and spherical tungsten carbide contribute to improved wear resistance due to their toughness.

The research work [19] recommends the hardfacing process using Ni-Cr-B-Si alloy on 316 LN stainless steel components in sodium-cooled fast reactors to enhance wear resistance and to prevent self-welding. The following three coating processes were comparatively analyzed: plasma transferred arc, TIG arc and laser.

Bembenek M. and colleagues [20] investigated the microstructure and mechanical properties of hardfacing alloys based on the Fe-Mo-B-C system. The obtained results show that the addition of Ti and Mn favor a significant increase in abrasion and impact–abrasion wear resistance by 1.2 and 1.3 times, respectively.

Gurumoorthy et al. [21] analyzed NiCrB alloy deposited on 316 LN SS substrate using the PTA process for nuclear applications. Aging studies were performed at 650 °C for 250 h on the deposited layer to estimate its microstructural stability. It was observed that the microstructure of the deposited layer, after artificial aging at 650 °C for 250 h, consisted of a solid solution γ, with the Ni base having a dendritic shape, and the Cr carbides and borides ensured good wear resistance.

Hemmati et al. [22] explained the role of Fe content in changing the microstructure of Colmonoy 69 layers deposited via laser cladding on S355 steel. It was observed that as the Fe content of the steel substrate increases to 25%, chromium borides with higher Fe fractions are formed. The precipitation of primary chromium borides is suppressed by increasing the Fe content above 40%. The Ni-B-Si eutectics led to a higher dilution. Due to these changes, the hardness decreased from 800 HV to 500 HV.

The present paper analyzes the parameters of the TIG welding deposition process using Corodur 65 electrode wire (EN 14,700 DIN 8555) on X2CrNiMoN22-5-3 duplex-stainless-steel substrates, having potential applications in the repairment of mechanical systems that work under conditions of mechanical wear and cavitation erosion. The parameters of the process are presented together with a diffusion analysis of the alloying elements from the base metal in the deposited metal, the hardness gradient and the macro- and microstructure of the layer–substrate system.

## 2. Experimental Procedure

The filler metal selected for the experiments was Corodur 65 (Corthal 65), DIN EN 14,700; its chemical composition is shown in Table 1.

This alloy was chosen because it has special characteristics in terms of high resistance to abrasive wear due to its chemical composition that combines a high content of C with that of Cr, Mo, Nb, W and V, maintaining a lower content of Fe. The base metal was stainless steel X2CrNiMoN22-5-3 (AISI 2205), which is used in the manufacturing of some components from the petrochemical industry, rotors and motor shafts of mining equipment, desalination plants, etc. Its chemical composition is displayed in Table 2.

**Figure 1:**
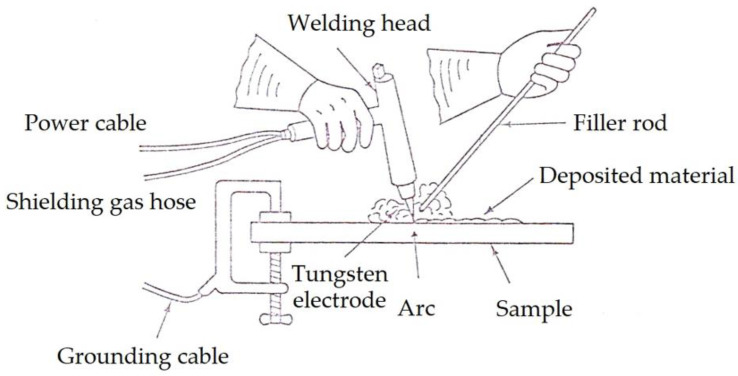
Schematic of the TIG welding process.

The TIG surface modification process provides a quality weld and a good metallurgical bonding between the deposited layer and the substrate. The intense heat of the electric arc progressively melts the base metal and the filler metal (Figure 1).

**Figure 2:**
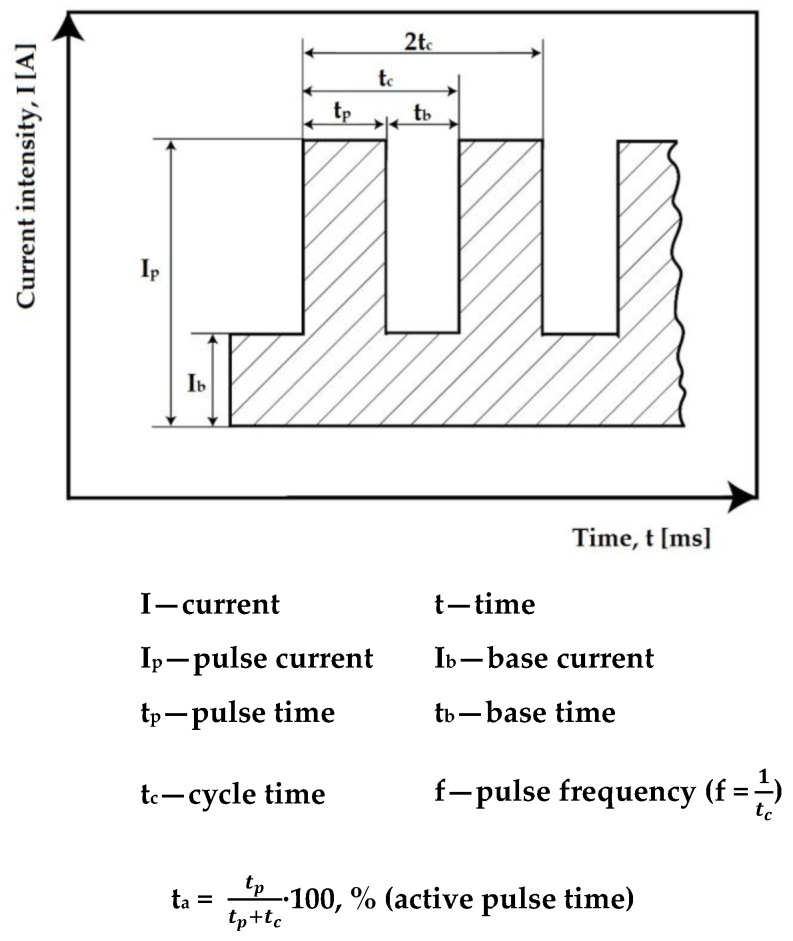
The parameters of pulsed current TIG welding process.

The technological variant in the pulsed current involves the rapid variation of the welding current between two levels, the higher current state, known as the pulse current (I_p_), and the lower current state, called the base current (I_b_), with a certain frequency. During the pulse current period, the welding area is heated and melted, and by lowering to the base current, the stable electric arc is maintained, and the cooling and partial crystallization of the molten metal pool occurs. Figure 2 describes the parameters of the deposition process via pulsed current TIG welding.

By properly adjusting the level of the specific parameters I_p_, I_b_, t_p_ and t_b_, respectively, and the frequency f, it is possible to precisely control the heat input introduced into the components. At the same average welding current, the seam penetration is greater than in classic TIG welding, and the heat input is lower, which leads to reduced welding stresses and deformations. As general recommendations, the pulse current has values that are 1.5–2 times higher than the classic constant current. The base current is usually taken as 25% of the pulse current, and the pulse time is between 0.02–1 s. depending on the welding technique. In practice, the so-called “one third rule” is frequently used according to which, to obtain an average current in the pulsed current that is equivalent to a constant reference current, the following values for the pulsed current are recommended:

I_p_ = 1.8 I_ref._

$$Ib=\frac{I_{p}}{3}\;(one\;third\;rule)$$

t_p_ = 0.2…0.3 s

t_b_ = 2·t_p_

In this case, the following is obtained:

I_ref._ = (I_p_·t_p_ + I_b_·t_b_)/(t_p_ + t_b_)

For example,

for I_ref._ = 50 A,

the results are as follows:

I_p_ = 90 A

$$Ib=\frac{I_{p}}{3}=30\;A$$

t_p_ = 0.3 s

t_b_ = 2·t_p_ = 0.6 s

I_m_ = I_ref._ = (I_p_·t_p_ + I_b_·t_b_)/(t_p_ + t_b_) = (90 × 0.3 + 30 × 0.6)/(0.3 + 0.6) = (27 + 18)/0.9 = 45/0.9 = 50 A

From the operator mode’s point of view, the welding is carried out as follows: the torch is held in place during the pulse time and is withdrawn during the base time, and the filler material is inserted during the pulse time and removed during the base time.

**Figure 3:**
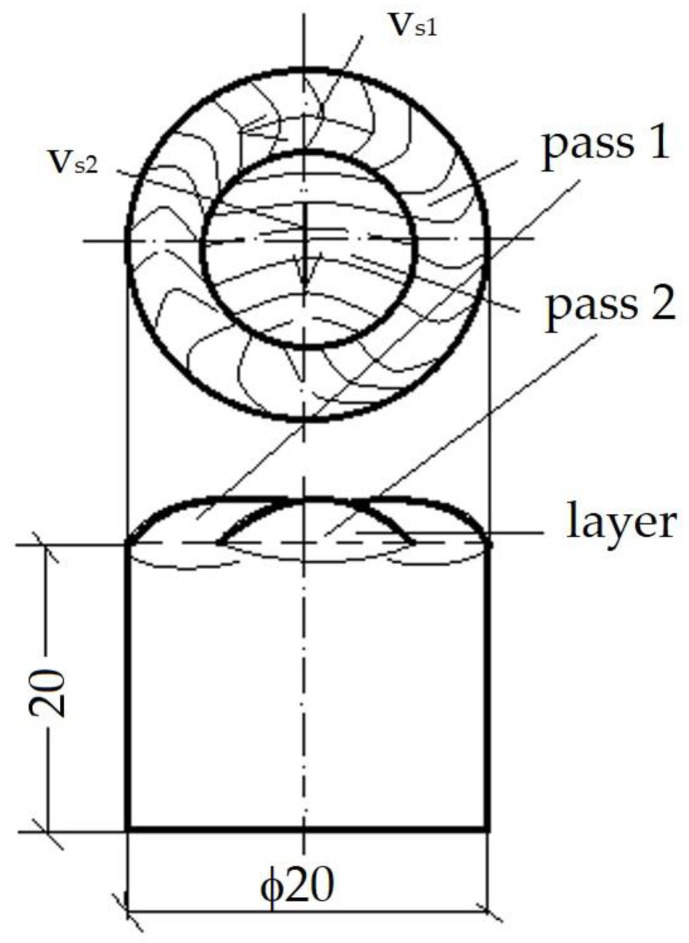
The order of layer deposition.

The experimental tests were carried out in the following welding conditions:

- Base metal—X2CrNiMoN22-5-3 (AISI 2205);
- Sample sizes—ø20 × 20 mm;
- Filler material—Corodur 65 (Corthal 65), DIN EN 14700;
- Type of filler material—tubular wire with self-protection;
- Wire diameter—1.6 mm;
- Shielding gas—Ar 100%, I1, EN 14,175 (Ar 4.8—LINDE);
- Shielding gas flow rate—10 l/min;
- Gas nozzle no. 7 (inner diameter 11.2 mm);
- Non-fusible electrode—EWC20 EN 26,848;
- Electrode diameter—2.4 mm;
- How to arrange the passes—on the frontal surface of the sample;
- Pass number—2 per layer, from outside to inside, as shown in Figure 3;
- Number of layers:
  ▪ Sample 1—1 layer;
  ▪ Sample 2—2 layers;
  ▪ Sample 3—3 layers.

It is specified that the selected wire is intended for welding deposition using the process with self-protection tubular wire. From this electrode, rods with dimensions ø1.6 × 200 mm were taken. The characteristic of this wire is that the deposition is free slag, making it suitable for TIG welding.

The process parameters were selected based on the results of several preliminary experiments and data obtained from the specialized literature. They have the following values:

- Impulse current—130 A;
- Base current—45 A;
- Average welding current—49 A; [I_m_ = (I_p_·t_p_ + I_b_·t_b_)/(t_p_ + t_b_)]—rectangular wave;
- Arc voltage—11 V;
- Pulse time—0.3 s;
- Base time—0.6 s (t_b_ = 2 t_p_);
- Pulse frequency—f = 1 Hz;
- Arc length—l_arc_ = 2.0–2.5 mm;
  ▪ Layer I, pass 1:
    - Welding time—10 s;
    - Weld length—43 mm;
    - Welding speed—26 cm/min;
    - Linear energy—2540 J/cm.
  ▪ Layer I, pass 2:
    - Welding time—5 s;
    - Weld length—10 mm;
    - Welding speed—12 cm/min;
    - Linear energy—5500 J/cm.
  ▪ Layer II, pass 1:
    - Welding time—8 s;
    - Weld length—40 mm;
    - Welding speed—30 cm/min;
    - Linear energy—2200 J/cm.
  ▪ Layer II, pass 2:
    - Welding time—4 s;
    - Weld length—10 mm;
    - Welding speed—15 cm/min;
    - Linear energy—4400 J/cm.
  ▪ Layer 3, pass 1:
    - Welding time—8 s;
    - Weld length—40 mm;
    - Welding speed—30 cm/min;
    - Linear energy—2420 J/cm.
  ▪ Layer III, pass 2:
    - Welding time—3 s;
    - Weld length—10 mm;
    - Welding speed—20 cm/min;
    - Linear energy—3300 J/cm.

**Figure 4:**
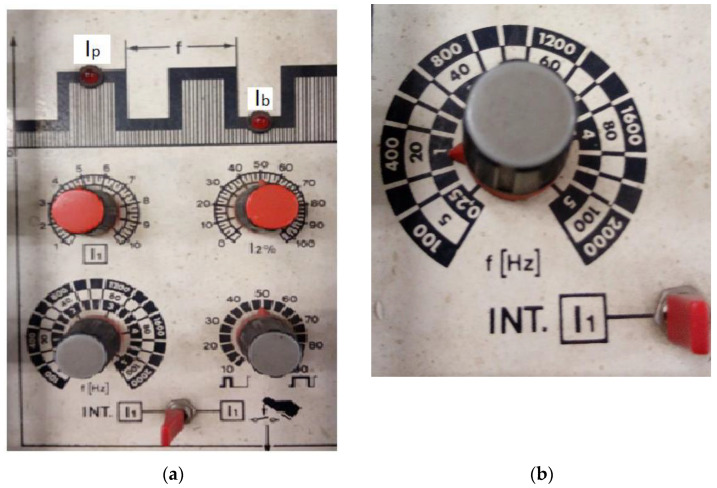
The settings of the used equipment: (**a**) tp = tb = 50% (lower right potentiometer); (**b**) f = 1 Hz.

Figure 4 shows the settings of the used welding equipment, namely, the source with inverter MW—300 from the Fronius company.

It can be seen that the welding parameters were kept constant except for the welding speed, which changed in value from one layer to another, respectively, from one pass to another, due to the sample’s heating conditions. It is specified that between the layers, each sample was allowed to cool down to a temperature of 100–150 °C in order to limit its overheating and to have a good control over the metal bath.

The choice of the pulse current (I_p_ = 130 A) was made according to the energy required for the stable melting of the rod, taking into account the chemical composition of the core, characterized by chemical elements with a high melting temperature. This involves the need for a higher heat delivered by the electric arc, specifically, the use of a welding current higher than that claimed by the 1.6 mm rod diameter. The distance between the electrode tip and the samples to be processed was 10 mm.

Although this value is unusual for conventional TIG welding, in the case of making multilayer welds, a previous study [23] showed that this distance ensures the least possible melting of the base metal without compromising the strong bond between it and the deposited metal. The distance from the wire tip to the samples to be processed was set at 3 mm to obtain a good correlation between the geometry of the welded joint and the dilution [24].

The dilution was maintained at acceptable values by changing the volume of the filler material introduced into the molten metal bath, which allowed for the regulation of the bath temperature and, therefore, the dilution with the base metal [25]. For example, when depositing the first layer, approx. 90 mm of filler material was used with 6 current pulses, which corresponds to 15 mm of molten rod at each pulse, which is relatively a lot for a 1.6 mm rod in normal welding conditions.

## 3. Evaluation of the Experimental Data

### 3.1. Chemical Heterogeneities and Degree of Dilution

For the chemical and metallographic analyses, the samples were cross sectioned followed by their conventional preparation; thus, after cutting, they were grounded with abrasive paper using silicon carbide and polished with diamond paste, and finally, they were etched via immersion for 30 s in the V_2_A reagent (20% hydrogen chloride, 5% nitric acid and 75% distilled water).

The dilution phenomenon is caused by the difference between the melting points of the base metal and the hardfacing alloy. The process of hard loading a substrate is accompanied by the penetration of its component elements into the deposited metal, affecting, to a certain degree, the properties of the hard coating alloy. Consequently, the properties of the alloy are diluted to a certain region at the interface, known as the dilution region. The evaluation of the dilution degree was made based on the chemical composition of the three distinct areas (base metal, deposited metal and filler metal) using an X-ray energy dispersion analysis. The calculation relationship is as follows [3]:

G_d_ = (Fe_wm_ − Fe_fm_)/(Fe_bm_ + Fe_fm_)·100 = (65.02 − 55.90)/(67.27 + 55.90) × 100 = 7.4%

where Fe_wm_, Fe_fm_ and Fe_bm_ represent the iron contents in weight percent of the weld metal, filler metal and base metal.

It is known that in the first moments of its formation, the molten metal bath is the place where important movements occur due to the thermal and dynamic action of the heat source. The superheated liquid undergoes a complex peripheral movement starting from the melting front to the rear area of the bath, through which a matter transfer occurs immediately. The duration of which the atoms are kept in the liquid phase is variable. Some of them are blocked by the solidification front movement, and others reach this front later, depending on the trajectory that is imposed on them. The rest of the atoms leave the environment in which they are via volatilization or chemical reactions. Consequently, the chemical composition of the molten zone varies both continuously and discontinuously.

Its continuous evolution is caused by the following [26]:

- Physical processes—volatilization of alloying elements;
- Chemical reactions between the components of the alloy;
- Reactions between the molten metal and the environment;
- Dilution;
- The fluctuation of the solidification speed.

Its discontinuous evolution is caused by the following [26]:

- Constitutional subordination;
- Dendritic segregation;
- Pollution;
- Irregular gas protection.

**Figure 5:**
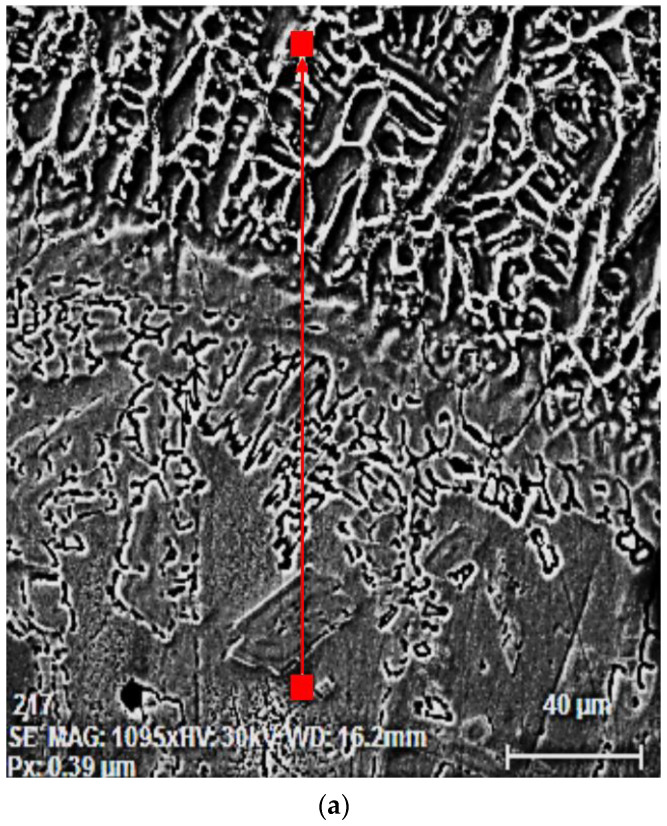

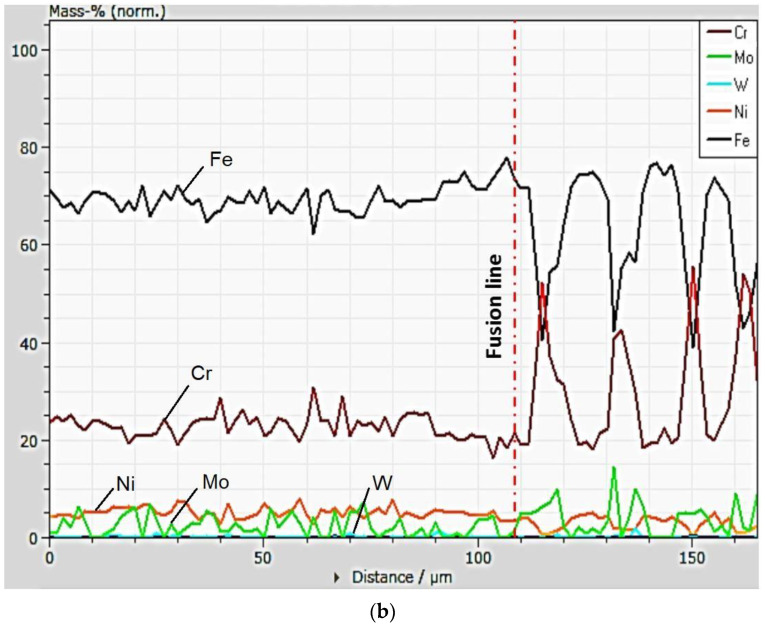
The linear variation of the concentrations of alloying elements in the region of the base metal–deposited metal interface (layer 1): (**a**) measurement direction; (**b**) evolution curves.

These aspects justify the changes in the chemical composition of the deposited metal layers from the Corodur 65 alloy. In Figure 5a, the measurement direction of the main alloying elements’ concentrations in the base metal and in the first zone of the dilution region is marked, and in Figure 5b, their linear variations are shown. If, in the base metal, there are limited variations in the concentrations of Fe, Cr, Ni and Mo, in the deposited metal, there are wide variations of Fe (from approx. 40% wt. to approx. 70% wt.), of Cr (from approx. 20% wt. to approx. 54% wt.), of Mo (from approx. 1% wt. to approx. 14.5% wt.) and of Ni (from 1 to 5% wt.) (Figure 5b).

**Figure 6:**
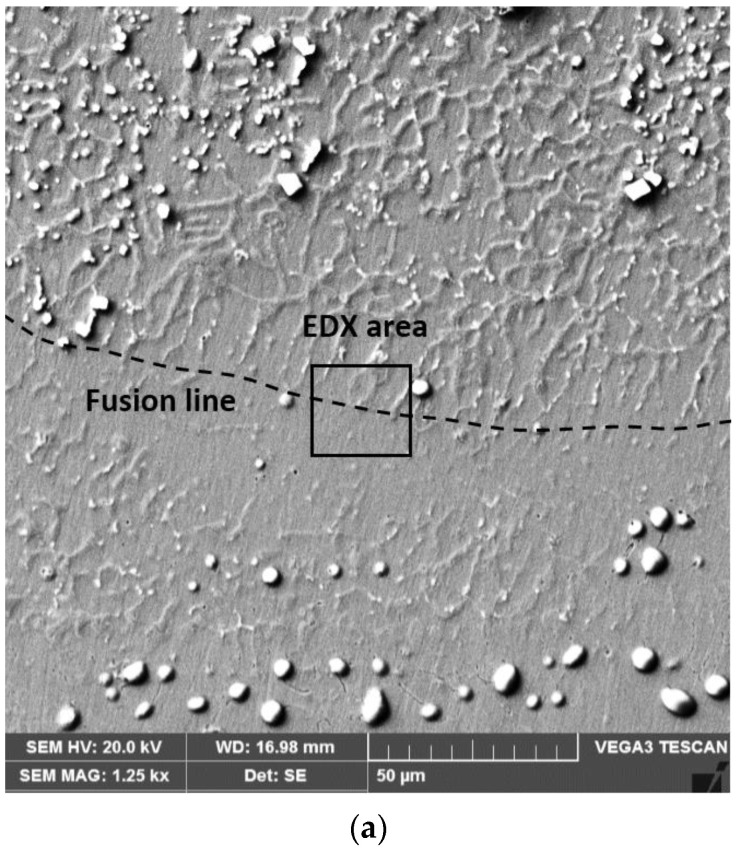

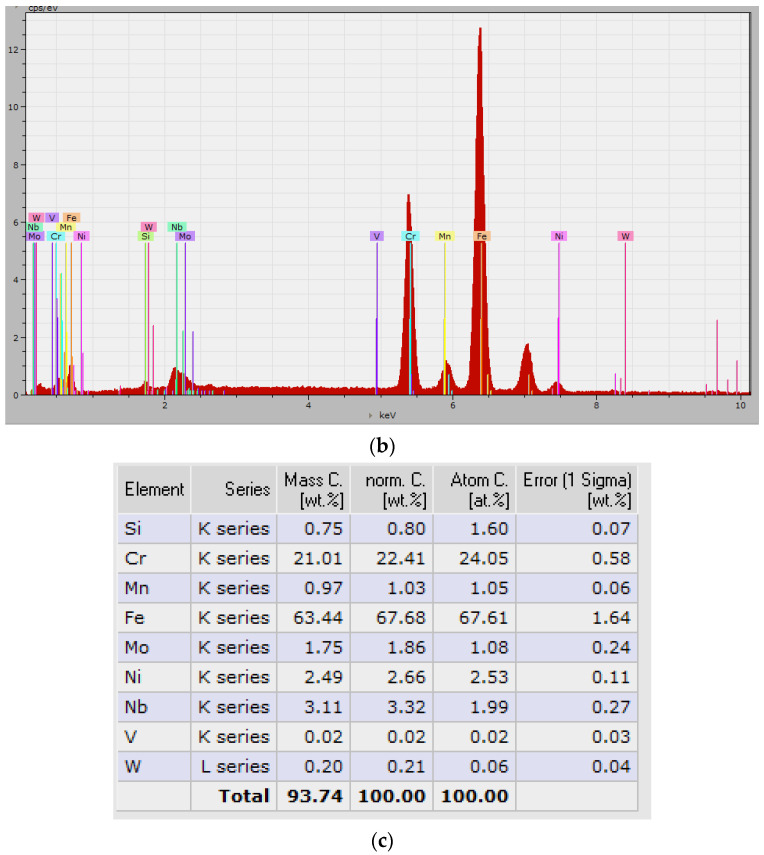
SEM micrograph (**a**), the energy dispersion spectrum of X-rays (**b**) and the chemical composition of a microregion from the base metal–deposited metal interface (**c**).

The X-ray energy dispersion analyses carried out on the areas of deposited metal in the immediate vicinity of the fusion line with the base metal (Figure 6) highlight important changes in the chemical composition determined by the volatilization phenomena of chemical elements, by the previously specified chemical reactions, dilution, etc.

**Figure 7:**
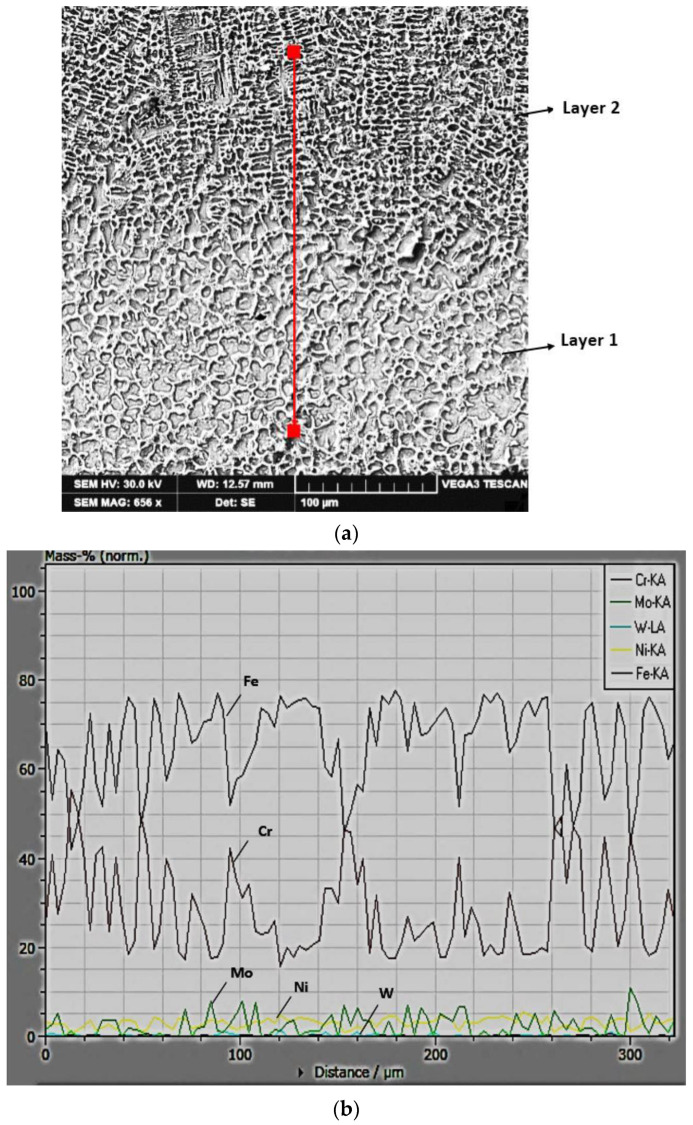
The microstructure (**a**) and the linear variation of the concentrations of alloying elements (**b**) in the region of the interface between layer 1 and layer 2 of the deposited metal.

During the molten metal bath solidification, the cooling rates specific to TIG welding are so high that they cause a segregation of the alloying elements. Since the alloy deposited via welding has a high content of Cr, which has a strong potential for segregation and is, at the same time, an element that forms complex carbides (FeCr)_23_C_6_, then one can expect wide variations in the concentrations of Fe and Cr in the entire volume of deposited metal (Figure 7).

**Figure 8:**
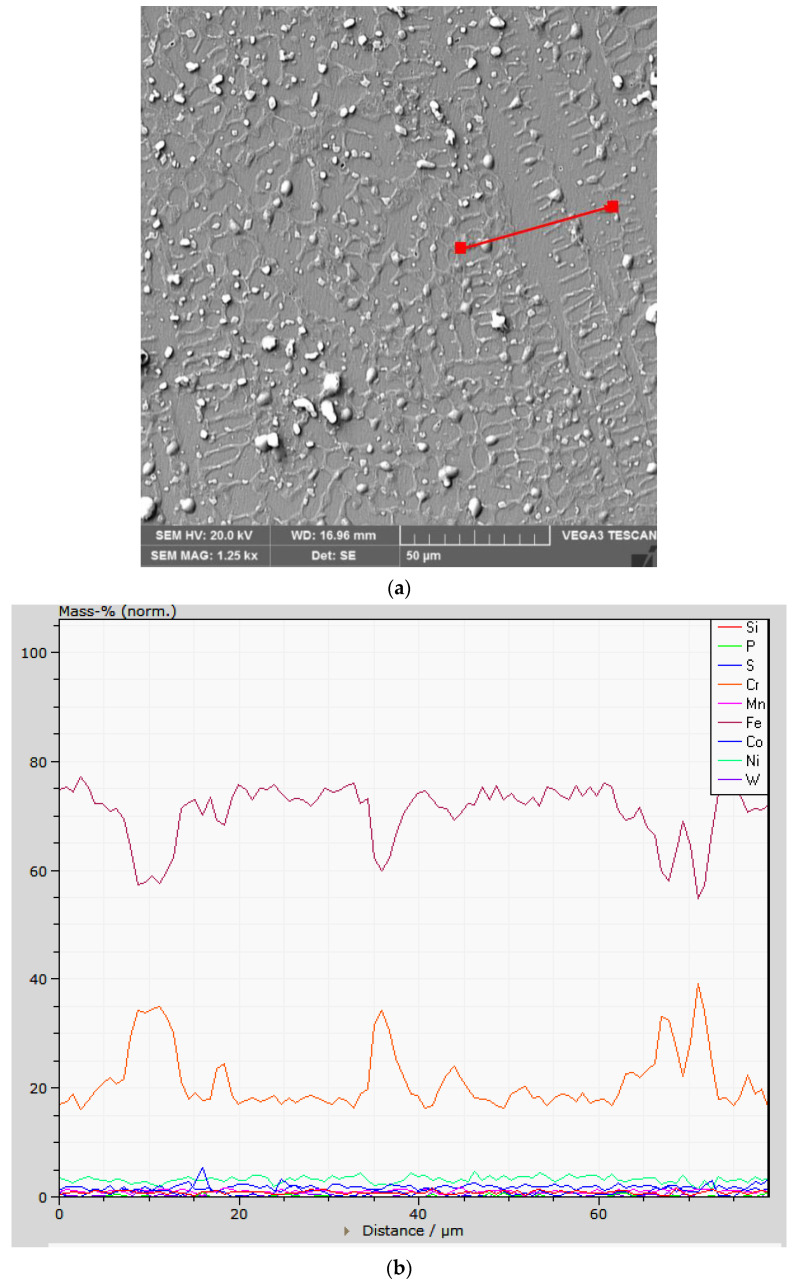
The microstructure (**a**) and the linear variation of the concentrations of alloying elements (**b**) in a cellular–dendritic region.

Figure 8 exemplifies the microstructure and the profile of the chemical composition in a direction transverse to that of cellular and dendritic growth. This shows an increase in the Cr concentration in the intercellular region, and for the same region, there is a depletion of Fe concentration.

### 3.2. The Microstructure of the Layer–Substrate System

A microscopic analysis provides the knowledge that allows for the quality evaluation of the deposited layers. The microstructural examinations were conducted using a Leica DM 2700 M optical microscope and a TESCAN VEGA 3 LMU Bruker scanning electron microscope equipped with an X-ray energy dispersive analysis system (EDX).

**Figure 9:**
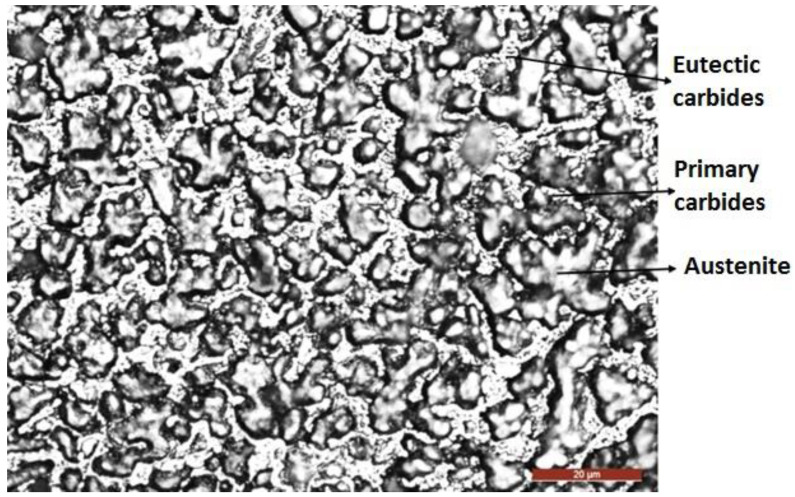
Optical microscopy (×500)—microstructure of the deposited metal.

**Figure 10:**
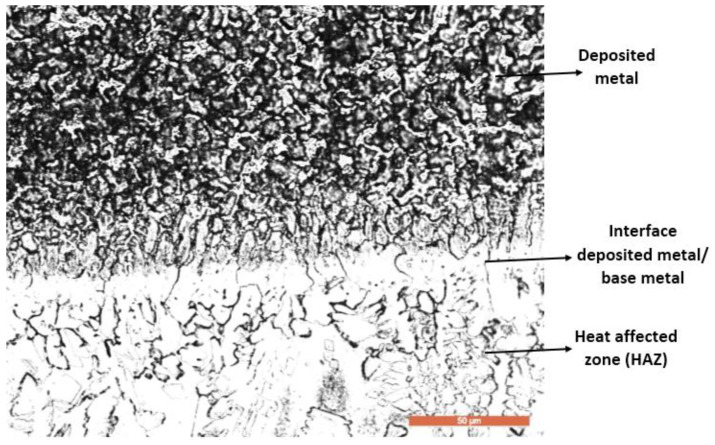
Optical microscopy (×500)—the microstructure of the interface between the deposited metal and the base metal.

**Figure 11:**
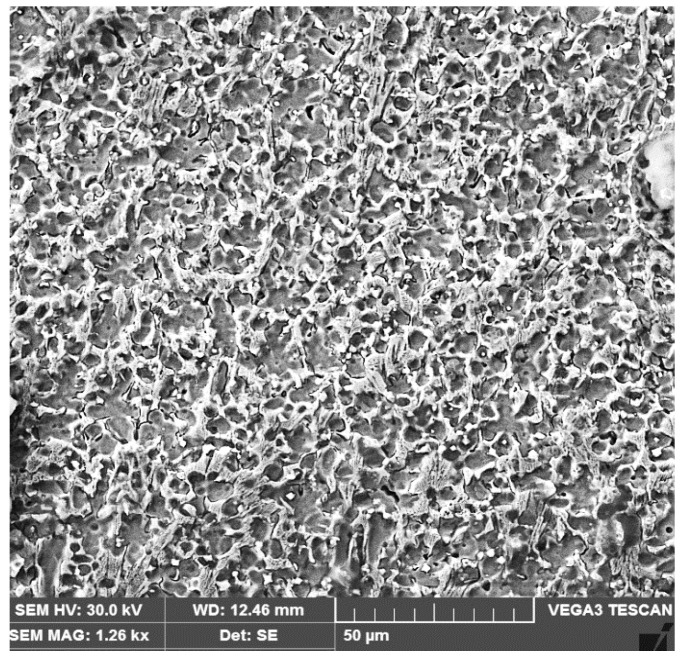
SEM micrograph (×1260) microstructure of the first deposited layer.

**Figure 12:**
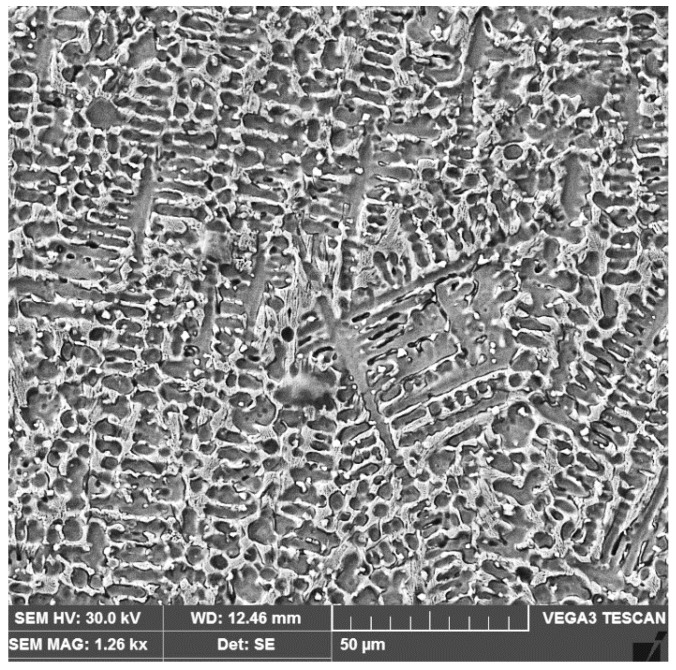
SEM micrograph (×1260) of the last deposited layer microstructure.

**Figure 13:**
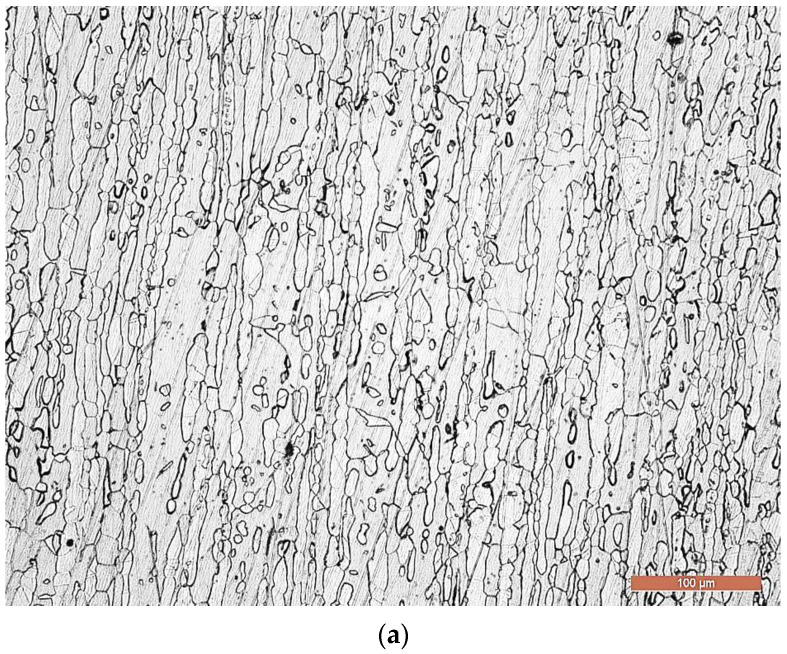

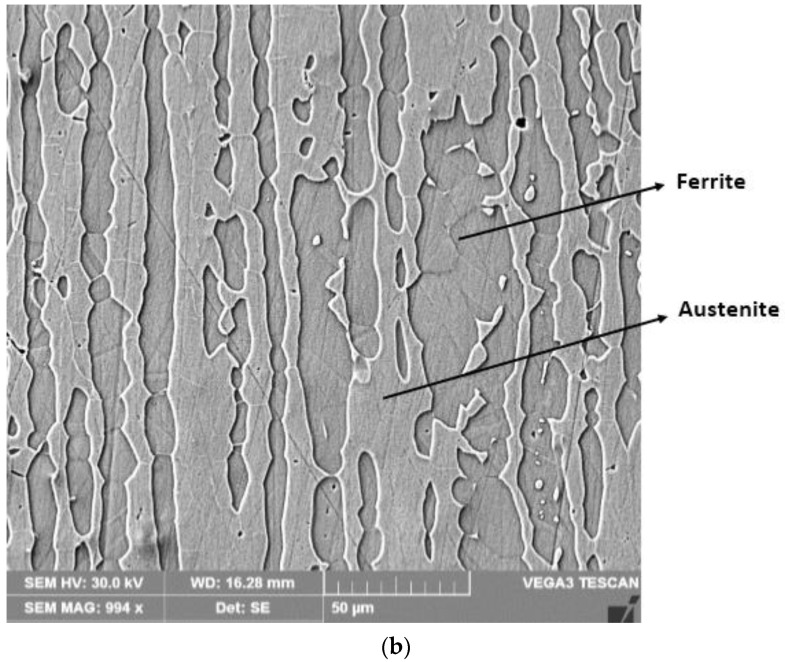
Microstructure of the BM: (**a**)—M.O. × 200; (**b**)—SEM × 1000.

Figure 9, Figure 10, Figure 11, Figure 12 and Figure 13 analyze the structural domains found in the deposited metal (DM), in the base metal (BM) and in the heat-affected influenced zone (HAZ) of the BM. Thus, in the deposited metal appears a microstructure composed of primary and eutectic Cr carbides, plus Nb-Mo-W-V carbides incorporated in an austenite matrix (Figure 9). These results are consistent with the existing data in other works from the specialized literature [27,28].

The investigations carried out using the scanning electron microscope highlight the fact that the secondary phases are precipitated both in the intercellular and interdendritic regions. The cell and cell-dendritic structures are located at the bottom of the fusion zone, immediately after the line of its interface with the base metal (Figure 11).

The interface between the first layer of the deposited metal and the heat-affected zone (HAZ) of the base metal contains a strip with a predominantly ferritic microstructure (Figure 10) without the appearance of cracking phenomena or other metallic continuity defects such as the lack of melting, pores, cracks and crevices. It is known that duplex stainless steels can tolerate relatively high linear energies and that their solidification structure is much more resistant to hot cracking than that of austenitic stainless-steel welds. However, if the linear energy is too high, the danger of forming a high proportion of intermetallic phases in the HAZ increases [3]. To avoid problems in the HAZ, the welding process must allow for a high-speed cooling of this region after welding. For these reasons, the maximum temperature between two successive passes was limited to 150 °C, as this temperature has the greatest effect on the HAZ cooling.

The growth of the dendritic zone comprises the largest part of the deposited metal volume. In the upper part of the fusion zone, a dendritic structure was also observed with a certain proportion of secondary phases that precipitated along all the interdendritic boundaries (Figure 12).

The base metal is delivered in the form of bars, sheets, pipes, wires, cast or forged pieces, which are either in a hyper-hardened state (solution treatment), or in a mechanically quenched state. It presents a microstructure consisting of approx. 52% austenite and 48% ferrite (determined using a Fischer ferritoscope) (Figure 13), which is typical for a duplex stainless steel. These results are in concordance with those found in other works from the specialized literature [3].

### 3.3. X-ray Diffraction Analyses

X-ray diffraction investigations were used to characterize secondary phases precipitated during solidification and/or solid-state transformations. The equipment used is the X Panalytical XPert Pro MPD spectrometer, and the X’Pert HighScore software and its database were used for post-processing and interference peak indexing. The scan details applied in these analyses were a 2Theta range from 20 to 100 degrees, with a step size of approximately 0.02 degrees, and counting at each step for 4 s. Cu Ka radiation was used, and the X-ray power generator was set at 45 kV and 30 mA.

**Figure 14:**
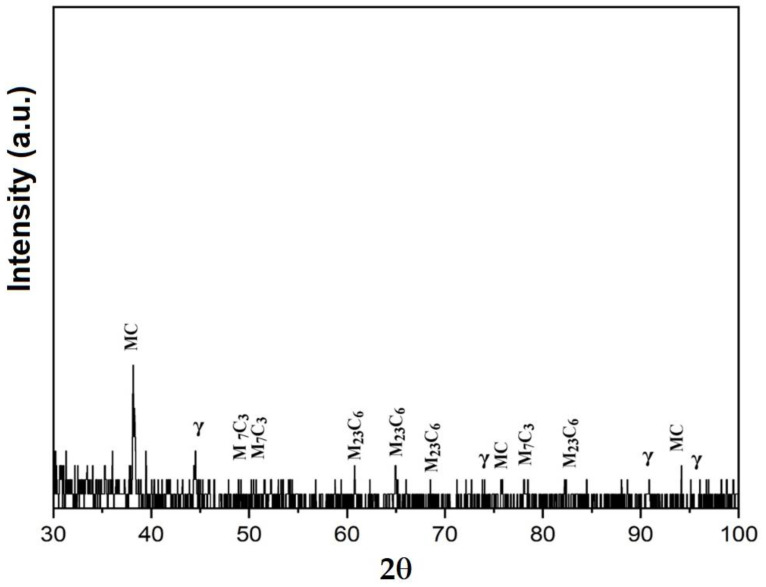
XRD pattern of the deposited metal Corodur 65.

**Figure 15:**
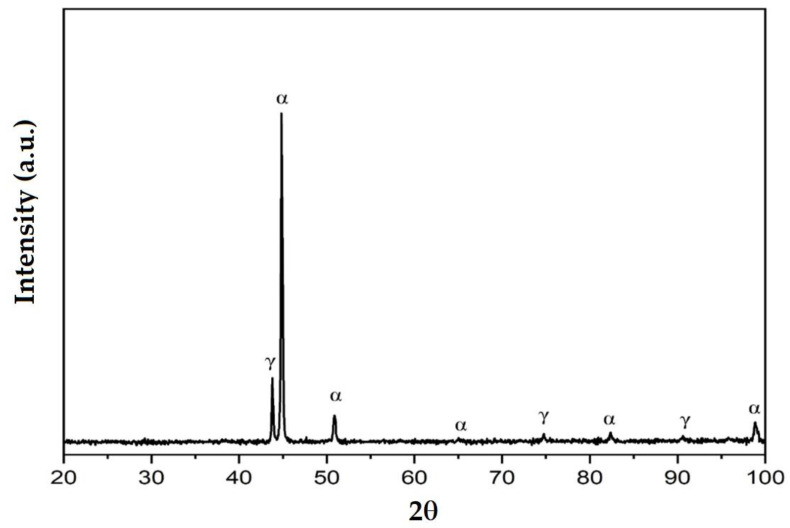
XRD pattern for the HAZ of the BM X2CrNiMo22-5-3.

**Figure 16:**
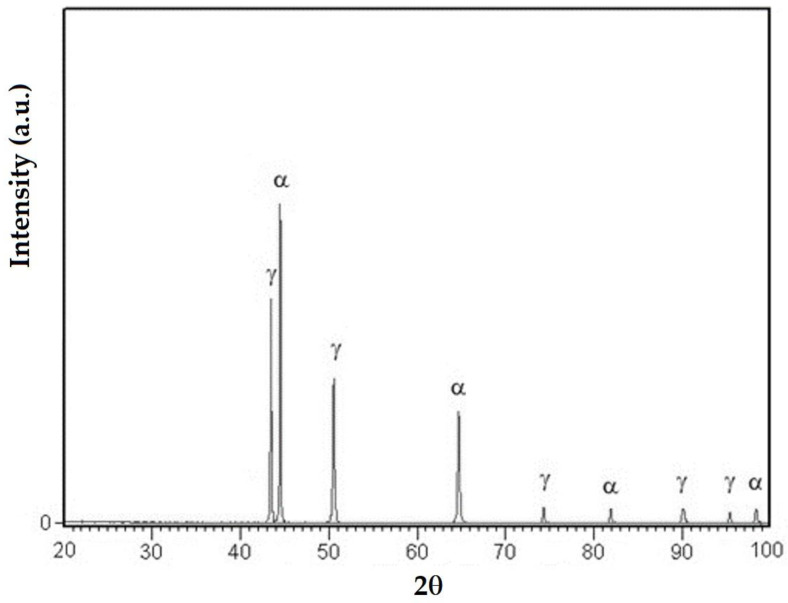
XRD pattern of the base metal X2CrNiMo22-5-3.

Figure 14, Figure 15 and Figure 16 exemplify the diffractograms of the deposited metal, the heat-affected zone of the base metal and the unaffected base metal via the heat welding cycle. Their analysis confirms the results of the microstructural examinations, highlighting the following aspects:

- The structural phases detected in the deposited metal are the particles of complex carbides dispersed in the solid solution γ with the fcc crystalline lattice.
- The high temperatures reached in the area adjacent to the fusion line promote, in the HAZ of the base metal, a predominantly ferritic microstructure with a reduced proportion of austenite resulting from the transformation into a solid state, initiated in the cooling stage of the deposited metal bath.
- The base metal in the solution treatment state has a microstructure made up of solid solutions, ferrite and austenite.

### 3.4. Hardness Measurements

**Figure 17:**
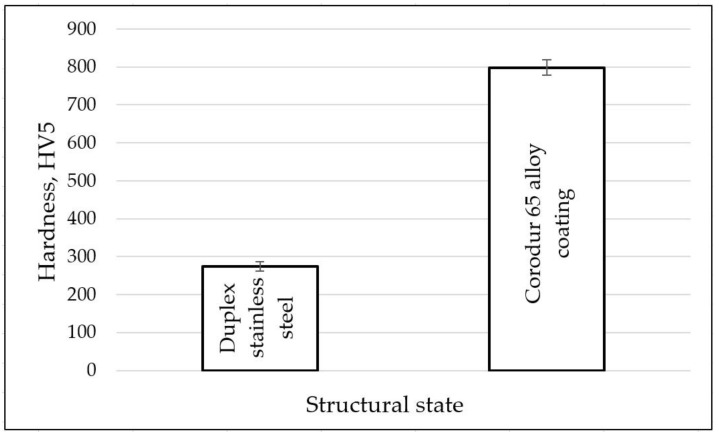
Hardness measurements of the investigated materials.

Since hardness is the most sensitive mechanical property to the structural changes in a metallic material, the samples coated with the Corodur 65 alloy and those from the solution-treated base metal were subjected to such examinations. On the front surface of these samples, seven Vickers hardness measurements were made with a load of 50 N/mm^2^ using the Zwick/Roell (YHV-S, Ulm, Germany) device. Based on the obtained values, the histogram shown in Figure 17 was drawn. The presented data demonstrate that there is a full agreement between the hardness and the microstructure of the layer–substrate system. The lowest hardness values are specific to duplex stainless steel (274–282 HV5). Instead, the hardfacing process using the Corodur 65 alloy ensures a high hardness (798–825 HV5), which favors a decrease in the wear rate. The high hardness values are attributed to the complex carbide particles in the layer deposited on the surface.

## 4. Conclusions

Based on the results obtained in this study regarding the microstructural characteristics of the Corodur 65 alloy layers deposited via TIG pulsed current welding on a duplex-stainless-steel substrate, X2CrNiMoN 22-5-3, the following conclusions are made:

- The most important process parameter is the welding current, as it influences the linear energy and the melting of the materials selected for the layer and for the substrate.
- The base metal was covered with three layers of the Corodur alloy—single, double and triple layer. The obtained hardness is remarkable, at approx. 810 HV5. Due to the low degree of dilution, there were no differences in the hardness between the deposited metal layers.
- The hardfacing process promotes, in the deposited layer, precipitation reactions of complex carbides finely dispersed in a tenacious austenite matrix.
- The precipitation of the carbide phases takes place both in the intercellular and interdendritic regions from the fusion zone located in close proximity to the base metal.
- The profile of the chemical composition in a direction transverse to that of cellular and dendritic growth indicates an increase in the Cr concentration and a decrease in the Fe concentration in the intercellular region.

## Figures and Tables

**Table 1:** Chemical composition of Corodur 65.

C%	Si%	Mn %	Cr%	Mo%	Nb%	V%	W%	Fe%
5.08	0.94	0.42	21.1	6.94	6.72	1.02	1.88	Rest

**Table 2:** Chemical composition of X2CrNiMoN22-5-3 stainless steel.

C%	Si%	Mn%	Cr%	Ni%	Mo%	P%	S%	N%	Fe%
0.017	0.72	1.80	22.08	5.02	2.90	0.021	0.012	0.16	Rest

## Data Availability

Not applicable.
